# New Insights into the Thermodynamic Properties and Raman Vibrational Modes of Polyhalite from Density Functional Theory

**DOI:** 10.3390/molecules31081269

**Published:** 2026-04-12

**Authors:** Huaide Cheng, Yugang Chen, Shichun Zhang

**Affiliations:** 1 Key Laboratory of Green and High-End Utilization of Salt Lake Resources, Qinghai Institute of Salt Lakes, Chinese Academy of Sciences, Xining 810008, China; chenyg@isl.ac.cn (Y.C.); zhangsc@isl.ac.cn (S.Z.); 2 Qinghai Provincial Key Laboratory of Geology and Environment of Salt Lakes, Xining 810008, China; 3 Qinghai Provincial Key Laboratory of Resources and Chemistry of Salt Lake, Xining 810008, China

**Keywords:** polyhalite, thermodynamic properties, Raman vibrational modes, DFT

## Abstract

Polyhalite, K_2_SO_4_•MgSO_4_•2CaSO_4_•2H_2_O, a ternary evaporite mineral, is commonly found in evaporitic rock salt strata, where it acts as an indicator mineral for potash evaporite deposits. As a directly exploitable mineral potash fertilizer, polyhalite serves as an important substitute for potassium resources. The thermodynamic properties of polyhalite remain poorly characterized experimentally; consequently, current estimates predominantly rely on predictive modeling and indirect experimental approaches. The Raman spectra of free SO_4_^2−^ vibrational modes in various sulfate minerals are sensitive to the local symmetry and hydrogen-bonding environment within crystal hydrates, and are directly influenced by the surrounding crystal field. This sensitivity makes Raman spectroscopy a powerful tool for investigating and identifying the crystal structures of sulfate minerals. In this work, the thermodynamic and Raman vibrational properties of polyhalite were investigated using density functional theory (DFT). Phonon calculations at the optimized geometry were employed to compute polyhalite’s key thermodynamic properties—specific heat, entropy, enthalpy, Gibbs free energy, and Debye temperature—over a temperature range of 0–1000 K. The results showed that: (1) the computed volume exhibited minimal error, approximately 0.87%, compared to experimental data; (2) the calculated values for the isobaric heat capacity and entropy were 420.72 and 531.39 J·mol^−1^·K^−1^ at 298.15 K, respectively; and (3) the calculated value for the free energy of formation at 298.15 K was −5670 kJ·mol^−1^. The computed Raman spectrum results showed that the typical spectral features of polyhalite are: (1) ν1 for 1024 cm^−1^, symmetric stretching mode; (2) ν2 for 464 cm^−1^, symmetry bending mode; and (3) ν4 for 627 cm^−1^, anti-symmetry bending mode.

## 1. Introduction

The mineral polyhalite, K_2_SO_4_•MgSO_4_•2CaSO_4_•2H_2_O, represents a ternary salt, which is widely distributed in evaporitic rock salt formations, with average contents ranging from 1% to 3%. It typically forms thin and multiple layers of lacustrine–lagoonal salt in the basin, resulting in a large cumulative thickness [1,2]. As a typical mineral of marine potash evaporite deposits, two main hypotheses have been proposed for its genesis: (1) the reaction between K^+^/Mg^2+^-enriched brine and early-deposited evaporites (gypsum, anhydrite or glauberite), and (2) direct precipitation from externally sourced supersaturated brines containing K^+^, Mg^2+^, Ca^2+^ and SO_4_^2−^ ions [3,4,5,6,7,8]. However, the physicochemical conditions and formation mechanism of polyhalite remain poorly understood. Although polyhalite can be used as a geological repository for storing nuclear waste [9], the heat accumulated from the radioactive decay of the nuclear waste stored at this site can decompose the hydrous phases [10]. In this case, its stability should play an important role and further research is needed. Geologically, polyhalite is used as a mineral geothermometer [11,12,13]. Commercially, polyhalite is mined as a source of potash, yielding chlorine-free potassium fertilizer and magnesium fertilizer, including potassium sulfate (SOP or K_2_SO_4_) and magnesium-bearing compounds such as leonite (K_2_SO_4_•MgSO_4_•4H_2_O), schoenite (K_2_SO_4_•MgSO_4_•6H_2_O), and langbeinite (K_2_SO_4_•2MgSO_4_), as well as magnesium sulfate like kieserite (MgSO_4_•H_2_O) and epsomite (MgSO_4_•7H_2_O) [14]. However, despite some investigations into the thermal analyses and genesis of polyhalite, its fundamental thermodynamic properties, such as the standard enthalpies, entropy, and Gibbs free energies of formation, remain scarcely studied. Therefore, accurate thermodynamic parameters are essential for assessing the genesis and evolutionary processes of potash deposits, in formulating effective mining strategies for polyhalite ore, and in the study of the dehydration process of polyhalite, and might also be significant in analyzing the phase transformation reactions of calcium-containing sulfate minerals.

Due to the scarcity of experimental data on the thermodynamic properties of polyhalite, estimations of these properties often rely on prediction and indirect methods. In an earlier study, the standard Gibbs free energy of polyhalite was estimated by using the values for the standard chemical potentials of the aqueous solution mineral [15]. In another study, the standard entropy was calculated through density functional theory (DFT) to determine the standard Gibbs free energy and the standard enthalpy of the formation of polyhalite [16]. The first enthalpy of formation data for natural polyhalite were obtained using a Calvet microcalorimeter [17]. The standard Gibbs free energy of formation of polyhalite was estimated using high-temperature drop-solution calorimetric measurements [9]. Weck and co-researchers [16] reported the thermodynamic properties of polyhalite up to 600 K. These scattered data are mainly concentrated in the standard state conditions, and thermodynamic data for polyhalite are still lacking in comprehensive databases of mineral properties [18].

Free SO_4_^2−^ has T_d_ symmetry. When incorporated into a crystal lattice, interactions with water molecules (via hydrogen bonding) and with cations reduce its local symmetry. This reduction lifts degeneracies and causes characteristic changes in the Raman spectrum: peak splitting, frequency shifts, and changes in relative intensity. Thus, the Raman spectrum serves as a fingerprint of the specific coordination environment and hydrogen bond network. This is why Raman spectroscopy is a key method for the structural analysis of sulfate minerals.

In this paper, the temperature dependence of different thermodynamic properties of polyhalite is investigated using density functional theory (DFT). We aim to obtain the thermodynamic properties of polyhalite across a wide temperature range of 0–1000 K, covering both the phase transformation range (620–800 K) and the temperature range relevant to nuclear waste disposal (300–800 K). In addition, the Raman vibrational properties of polyhalite are investigated based on DFT calculations.

## 2. Results and Discussion

### 2.1. Crystal Structures

**Figure 1:**
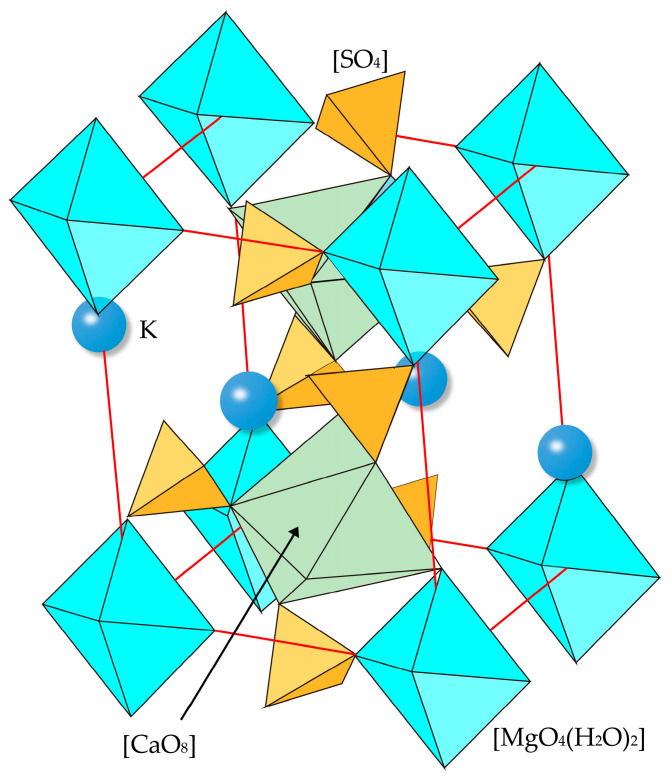
Crystal structure schematic of polyhalite. Tetrahedra represent [SO_4_] units, octahedral represent [MgO_4_(H_2_O)_2_] units, polyhedra represent [CaO_8_] units and spheres represent K^+^ cations.

The structure model of polyhalite was first studied by Schlatti et al. [19], revealing that (1) SO_4_ groups are connected via eight-coordinated Mg, eight-coordinated Ca, and eleven-coordinated K; (2) each H_2_O molecule is coordinated by one Mg and one K; and (3) the probable position of the H-atoms is inferred from the interatomic distances. In 2005, its structure was reinvestigated by Bindi [20], indicating that (1) the framework comprises edge- and face-sharing polyhedra of K/Ca coordinated units with [MgO_4_(H_2_O)_2_] polyhedra, and (2) insular SO_4_ tetrahedra connect to these [MO_x_] polyhedra (M = K, Ca, Mg) through edge-sharing configurations (See Figure 1). After that, a DFT investigation into the structure of polyhalite was carried out by Weck et al. [16]. However, the error in the computed volume with respect to that of experimental data obtained by Bindi [20] is about 5%. In this paper, the lattice parameters of polyhalite as well as the volume were determined through calculations with increasing kinetic energy cutoffs and k-point mesh parameters.

The polyhalite optimized lattice parameters and volume are presented in Table 1 alongside the corresponding experimental results. Additionally, the DFT calculations of lattice parameters and volume reported by Weck et al. [16] are included in this table. The error in the computed volume with respect to that of experimental data obtained by Bindi [20] is very small, about 0.87%, while the error of a DFT calculation volume published by Weck et al. [16] is about 4.1%.

For the polyhedra of polyhalite, the predicted K-O and Ca-O bond distances range from 2.61 to 3.36 Å and 2.29–2.68 Å respectively, with Mg-O distances of 2.05–2.34 Å. The S-O bond distances in the tetrahedra span the range 1.46–1.61 Å. Experimentally, Bindi [20] documented distinct bond distance ranges: K-O bonds (2.77–3.20 Å), Ca-O bonds (2.40–2.69 Å), Mg-O bonds (2.02–2.16 Å), and S-O bonds exhibiting the shortest distances at 1.46–1.50 Å. Theoretical calculations by Weck et al. [16] determined the following bond distance ranges: 2.78–3.36 Å for K-O, 2.41–2.65 Å for Ca-O, 2.04–2.19 Å for Mg-O, and 1.48–1.51 Å for S-O bonds. More details on band distances are provided in Table S1 of the Supplementary Materials. As is well known, the bond distance reflects the magnitude of its bond energy. Based on the bond lengths and ionic charges, the relative bond strength is expected to decrease in the order S-O > Mg-O > Ca-O > K-O, which is consistent with the increasing ionic radius and decreasing charge density of the cations. However, a full assessment of bond stability would also require consideration of coordination environments and the degree of covalency.

**Figure 2:**
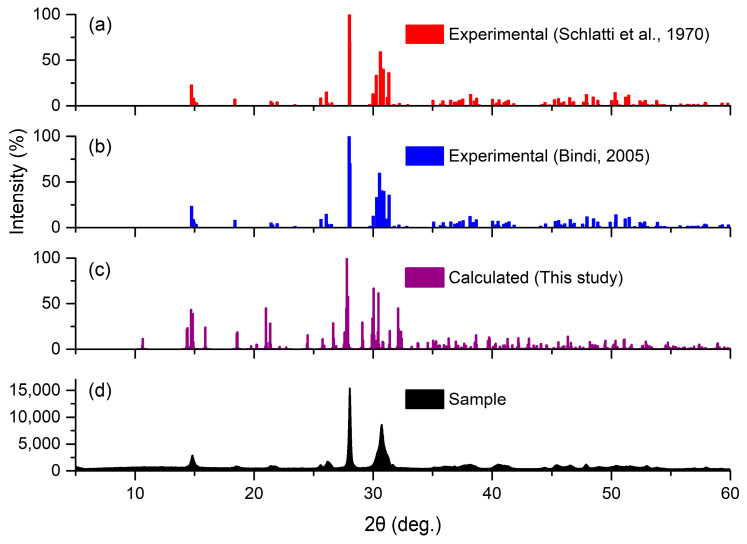
X-ray powder pattern diffractogram of polyhalite obtained using CuK_α_ radiation: (**a**) X-ray powder experimental pattern obtained by Schlatti et al. [19]; (**b**) X-ray powder experimental pattern obtained by Bindi [20]; (**c**) X-ray powder pattern computed in this work; and (**d**) X-ray powder experimental pattern from the sample, which was collected at Kunteyi Playa in Qinghai, China.

The computed pattern, alongside experimental results, is presented in Figure 2. Additionally, the experimental X-ray powder pattern from a sample collected at Kunteyi Playa in Qinghai, China, which is a kind of modern salt lake sedimentary type, is included in Figure 2. The agreement between the computed and experimental patterns is largely consistent, with notable correspondence in both line positions and intensities. A detailed comparison of the computed and experimental X-ray powder patterns of polyhalite is given in Table S2 and Figure S1 of the Supplementary Materials.

### 2.2. Thermodynamic Properties

A phonon calculation was performed at the optimized structure of polyhalite. Using these data, the thermodynamic properties were evaluated. The calculated heat capacity and entropy are presented in Figures S2 and S3 of the Supplementary Materials, respectively, based on the CASTEP module. The coefficients of the isobaric heat capacity, as shown in Equation (6), are provided in Table 2 as determined using a nonlinear least-squares regression method with a Hass–Fisher-type polynomial. More details on the calculations and data fitting are given in Table S3 and Figure S4 of the Supplementary Materials.

**Figure 3:**
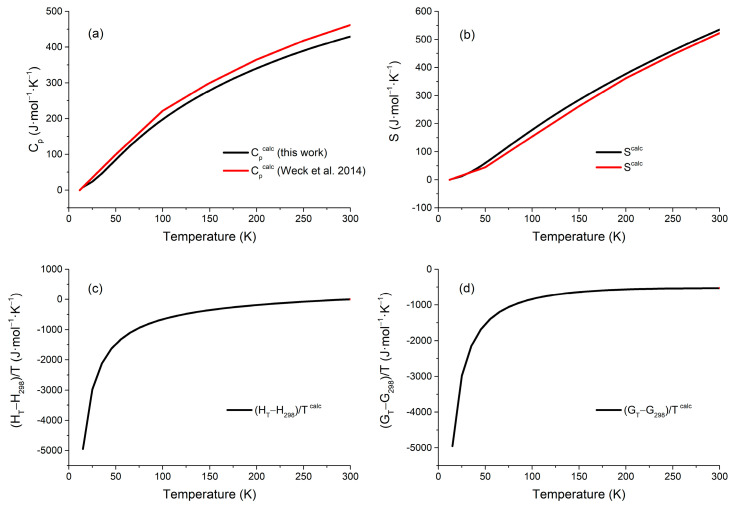
Calculated low-temperature thermodynamic properties of polyhalite: (**a**) isobaric heat capacity; (**b**) entropy; (**c**) enthalpy; and (**d**) Gibbs free energy. The calculated data from Weck et al. [16] are given.

The thermodynamic functions of polyhalite calculated over the temperature range of 0–1000 K are provided in Tables S3–S6 of the Supplementary Materials. Figure 3a–d respectively display the computed thermodynamic functions including isobaric heat capacity, entropy, enthalpy, and Gibbs free energy at low temperatures, with comparative analysis against the reference data reported by Weck et al. [16] within the temperature range of 0–300 K. The calculated isobaric heat capacity shows good agreement with Weck et al. [16], but deviations increase with temperature, reaching a maximum of 7% at 300 K. Since the thermodynamic properties of polyhalite have not been determined experimentally, its values were predicted or estimated. In this work, the predicted value of the isobaric heat capacity and entropy at 298.15 K are $C_{P}$ = 420.72 J·mol^−1^·K^−1^ and S = 531.39 J·mol^−1^·K^−1^. The calculated value of the entropy is lower than the values of Ogorodova et al. [17], S = 599.1 ± 1.3 J·mol^−1^·K^−1^, which is estimated via a reaction of minerals with known S values, the difference being about 11%. The value of entropy obtained by Guo and Xu [9] is S = 575.4 ± 12.4 J·mol^−1^·K^−1^, which is estimated based on the S-V relation (V is the unit-cell volume) for ionic-hydrate solids, the error being 7.6%. Weck et al. [16] gave a value of entropy of S = 518.55 J·mol^−1^·K^−1^, which is in best agreement with our value, the error being 2.4%.

These discrepancies are not unexpected, given the absence of direct experimental thermodynamic measurements for polyhalite. The values from refs. [9,17] are based on empirical estimations or semi-empirical correlations, which may involve inherent uncertainties and assumptions. By contrast, our calculation is derived from first-principles phonon calculations using the high-precision single-crystal structure of Bindi et al. [20]. The reliability of our computational approach is supported by the excellent agreement between the optimized lattice parameters and the experimental data, with a deviation of only 0.87% in unit-cell volume—a significant improvement over previous DFT calculations (Weck et al. [16], 4.1%). This level of structural accuracy, achieved using norm-conserving pseudopotentials with a high kinetic energy cutoff (800 eV) and dense k-point sampling (3 × 3 × 2), along with the inclusion of Grimme’s D3 dispersion correction to account for van der Waals interactions, provides confidence that the thermodynamic properties predicted in this work are reliable. Overall, the calculated entropy function is in very good agreement with that computed by Weck et al. [16], in the range 0–300 K, and the deviations from other estimations reflect the inherent variability among different predictive approaches in the absence of direct experimental data.

**Figure 4:**
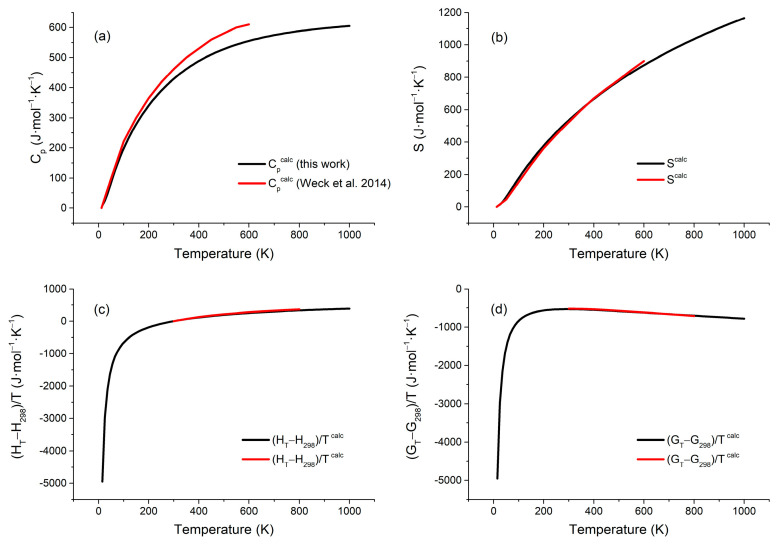
Calculated high-temperature thermodynamic properties of polyhalite: (**a**) isobaric heat capacity; (**b**) entropy; (**c**) enthalpy; and (**d**) Gibbs free energy. The calculated data from Weck et al. [16] are given.

Figure 4a–d present the calculated isobaric heat capacity, entropy, enthalpy, and Gibbs free energy functions at elevated temperatures, with comparative data derived from Weck et al.’s computations [16]. As can be seen from Figure 3, the predicted enthalpy and Gibbs free energy functions are in very good agreement with previous research by Weck et al. [16], in the range 300–1000 K. The predicted isobaric heat capacity function is lower than the data of Weck et al. [16], and there are still slight deviations between them. This deviation increases with temperature increases, with a maximum deviation of 10% at 600 K. The calculated values of entropy, enthalpy, and Gibbs free energy at specified temperatures are presented in Table S7 of the Supplementary Materials.

**Figure 5:**
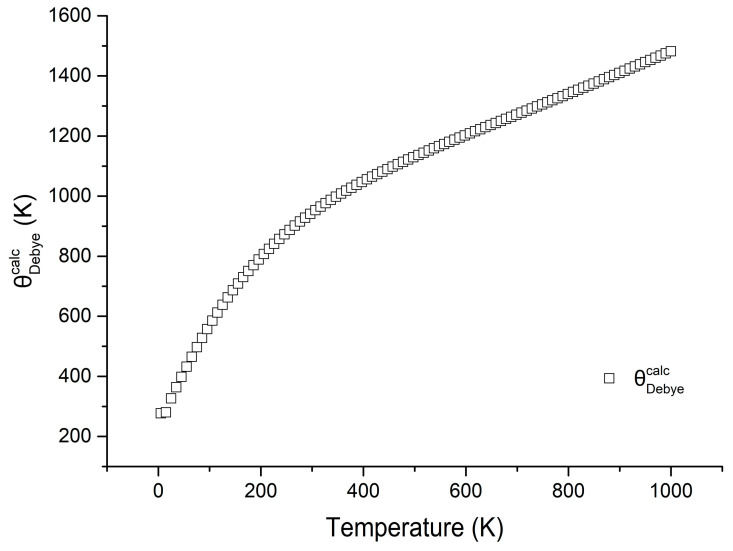
Calculated Debye temperature of polyhalite.

As a fundamental physical parameter, the Debye temperature (Θ_Debye_) serves as a critical indicator for evaluating structural stability and chemical bonding strength in materials. Furthermore, it exhibits strong correlations with essential thermodynamic properties including specific heat capacity and melting point. Figure 5 shows the calculated Debye temperature function of polyhalite.

### 2.3. Enthalpies and Free Energies of Formation

**Figure 6:**
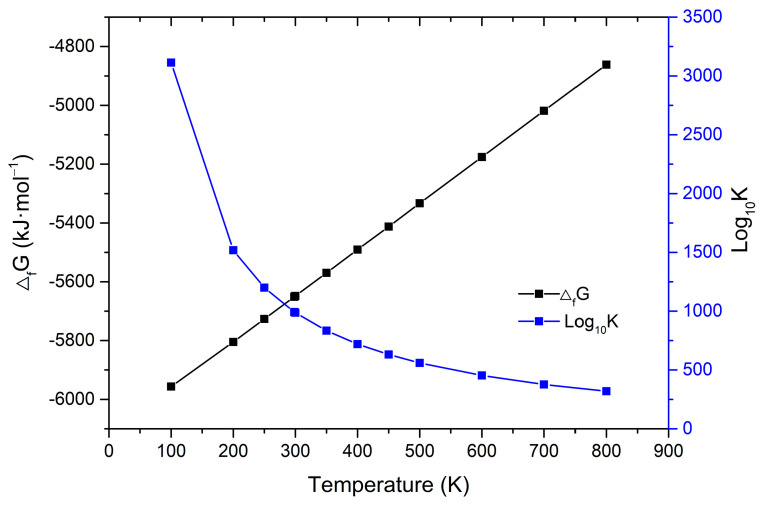
Calculated free energy of formation and reaction constant of polyhalite as a function of temperature.

The precise value of the standard state enthalpy of formation employed in our computations is the one from previous research by Ogorodova et al. [17], $\Delta_{f}H^{0}$ = −6121 ± 21 kJ·mol^−1^. The results obtained for the enthalpies, free energies of formation, and reaction constant of polyhalite at selected temperatures are given in Table S8 of the Supplementary Materials. The results are also displayed in Figure 6. Based on the calculated thermodynamic properties of polyhalite, the standard Gibbs free energy of formation (at 298.15 K) was determined to be $\Delta_{f}G^{0}$ = −5670 kJ·mol^−1^. This is in good agreement with the experimental value provided by Harvie et al. [15], $\Delta_{f}G^{0}$ = −5658.18 kJ·mol^−1^, with a deviation of 0.21%. The obtained $\Delta_{f}G^{0}$ value is also in general agreement with those determined by other experiments, $\Delta_{f}G^{0}$ = −5559 ± 21 kJ·mol^−1^ by Ogorodova et al. [17] and $\Delta_{f}G^{0}$ = −5739.3 ± 9.9 kJ·mol^−1^ by Guo and Xu [9]. In addition, the obtained $\Delta_{f}G^{0}$ value is in good agreement with the calculated value based on a certain assumption by Risacher and Fritz [21], $\Delta_{f}G^{0}$ = −5659.07 kJ·mol^−1^.

### 2.4. Raman Spectrum

**Figure 7:**
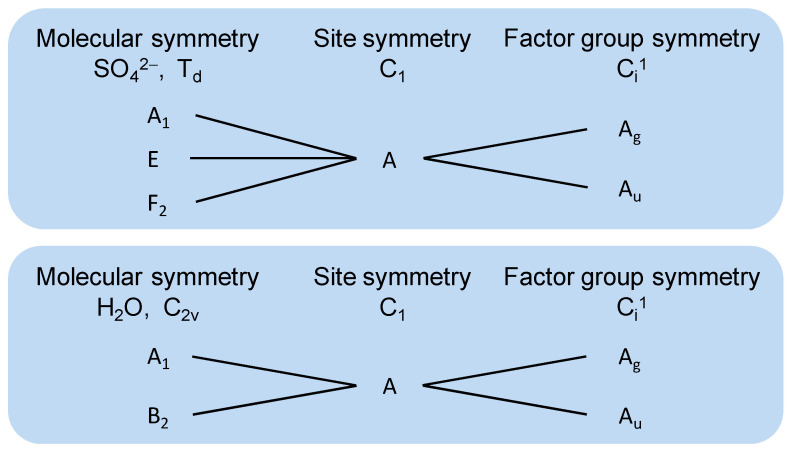
Correlation scheme for the vibrational modes of SO_4_^2−^ and H_2_O in polyhalite.

Polyhalite adopts the triclinic space group $P\bar{1}$ ($C_{i}^{1}$), with one formula unit per unit cell [20]. All constituent ions and water molecules (excluding Mg^2+^) occupy general positions within the lattice structure. The Mg^2+^ ions are specifically located at a crystallographic site exhibiting $C_{i}$ symmetry [22]. The correlation of the fundamental modes of the SO_4_^2−^ ion (T_d_ point group) and the H_2_O molecule (C_2v_ point group) with the C_1_ symmetry of their respective sites and the unit-cell symmetry C_i_ is illustrated in Figure 7.

As shown in Figure 7, the SO_4_^2−^ ions (with T_d_ point group symmetry) occupy crystallographic sites of lower C_2v_ symmetry. This symmetry reduction completely lifts the degeneracy of free-ion vibrational modes. The noncentrosymmetric nature of the primitive unit cell further causes each vibrational mode to split into two components: even (g) and odd (u) modes with respect to inversion symmetry. According to the mutual exclusion principle, the g-modes are exclusively Raman-active while the u-modes exhibit infrared activity.

Using the Correlation Method [23], polyhalite is expected to exhibit 93 fundamental modes, including the acoustic modes, which are split into:

(1) $${Г}_{93}=45A_{g}+48A_{u}$$

On that basis, the four degenerate vibrational modes of free tetrahedral SO_4_^2−^ (belonging to T_d_ point group symmetry) are split into 18 distinct Raman-active modes through factor group interactions: 2 for ν_1_ symmetric stretching, 4 for ν_2_ bending, 6 for ν_3_ anti-symmetric stretching, and 6 for ν_4_ anti-symmetric bending. Stretching modes typically occur in the 950–1200 cm^−1^ range, while bending modes appear in the 400–650 cm^−1^ range. A comparison of the experimental and calculated spectra is provided in Table 3. Although all SO_4_^2^ ions occupy general positions, the Raman spectrum of polyhalite displays two distinct bands at 1014 and 987 cm^−1^, corresponding to the ν_1_ modes of Ag symmetry [24]. Previous research on free SO_4_^2−^ ions in polyhalite [14] have demonstrated characteristic vibrational modes at: 1018 cm^−1^ for the symmetric stretching mode (ν_1_), 464 cm^−1^ for symmetry bending (ν_2_), and 630 cm^−1^ for anti-symmetry bending (ν_4_). Additional signals for free SO_4_^2−^ ions were observed at 1165, 1135, and 1074 cm^−1^ for ν_3_; 630 and 622 cm^−1^ for ν_4_; and 468, 440, and 455 cm^−1^ for ν_2_. This multiplicity of bands likely arises from the presence of crystallographically distinct sulfate tetrahedra within the polyhalite structure, each experiencing slightly different local coordination environments. Specifically, the sulfate groups differ in their interactions with adjacent cations and in the hydrogen-bonding networks formed with the structural water molecules, leading to subtle variations in the vibrational frequencies. These experimental values align well with the calculated results from this study’s Raman spectral analysis (See Table 3).

**Figure 8:**
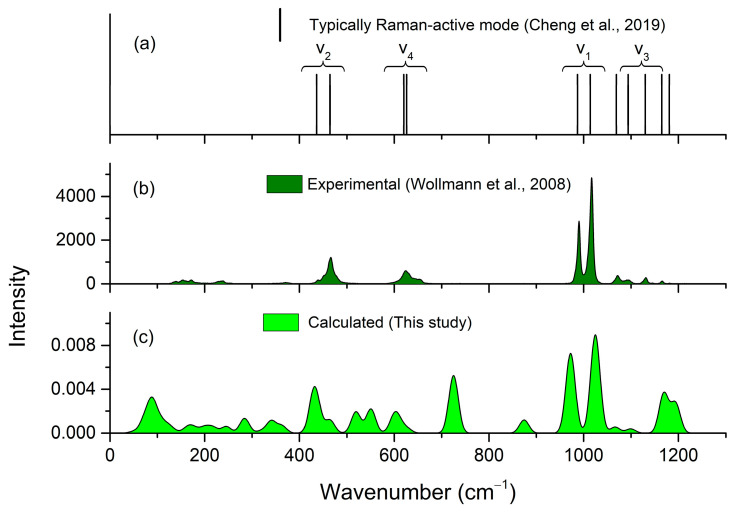
Raman spectra of polyhalite: (**a**) typically Raman-active mode [14]; (**b**) experimental data obtained by Wollmann et al. [24]; and (**c**) calculated Raman spectra for lattice in this study.

Figure 8 shows the calculated Raman spectra for the correlation for lattice and internal (v_1_, v_2_, v_3_, v_4_) modes of polyhalite, compared with the experimental data obtained by Wollmann et al. [24]. As shown in Figure 8, the computed spectrum is overall consistent with the experimental results, especially the typically Raman-active mode, v_1_, v_2_, v_3_, v_4_.

**Figure 9:**
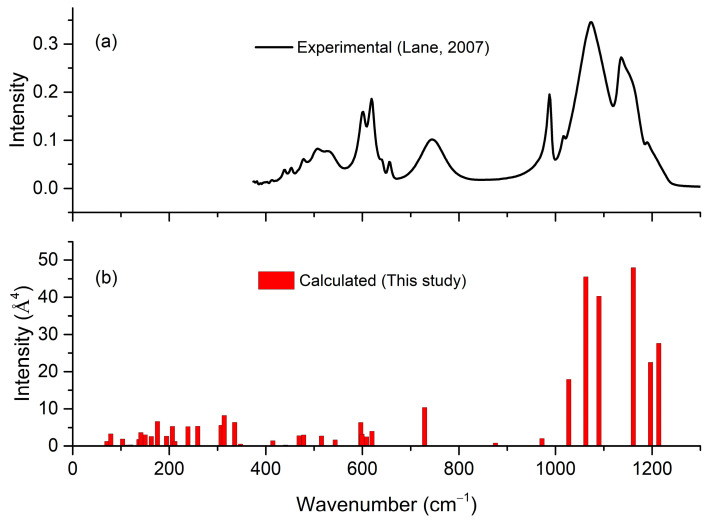
Infrared spectra of polyhalite: (**a**) experimental data of Lane Raman spectra for lattice in this study [25], and (**b**) calculated typical infrared-active shift and intensity.

The calculated infrared spectra of polyhalite are also given in Table 4. Figure 9 shows the calculated infrared shits of polyhalite, compared with the experimental data obtained by Lane [25]. As illustrated in Figure 8, there is good agreement between the calculated and experimental patterns, indicating that the bending and stretching vibrational modes predominantly occur in the 400–1200 cm^−1^ range.

## 3. Computational Methods

In this study, all computations were performed using the CASTEP module of Material Studio software, version 8.0. [26], which was an implementation of the pseudopotential plane-wave method, based on density functional theory (DFT). The crystallographic data of polyhalite from the American Mineralogist Crystal Structure Database was adopted and utilized as input files [27]. The generalized gradient approximation (GGA) [28] together with the Perdew–Burke–Ernzerhof (PBE) [29] functional and Grimme empirical dispersion corrections, called the DFT-D approach, were chosen to determine the exchange-correlation energy [30]. The pseudopotentials used for all the atoms were standard norm-conserving pseudopotentials [31]. Geometry optimization was performed using the Broyden–Fletcher–Goldfarb–Shanno (BFGS) [32] algorithm with a force convergence criterion of 0.01 eV/Å. To ensure computational accuracy, the kinetic energy cutoff [33] and k-point mesh [34] were systematically tested for energy convergence. Geometric optimization was carried out by using the following convergence criteria: (1) maximum atomic forces below 0.03 eV/Å, (2) maximum stress under 0.05 GPa, and (3) maximum atomic displacement within 0.001 Å. Additionally, the total energy and self-consistent field (SCF) calculations were converged to 1.0 × 10^−5^ eV/atom and 2.0 × 10^−6^ eV/atom respectively. All atomic orbitals of polyhalite involved in the calculation were treated as Ca(3p^6^, 4s^2^), K(3p^6^, 4s^1^), Mg(2p^6^, 3s^2^), O(2s^2^, 2p^4^), S(3s^2^, 3p^4^), and H(1s^1^), respectively. The structure of polyhalite was optimized by systematically increasing the values of the kinetic energy cutoff and k-point mesh parameters. The optimization performed with a cutoff of 800 eV and k-point scheme of 3 × 3 × 2 for polyhalite gave well converged structures and was therefore used to determine the final results.

The thermodynamic properties of polyhalite, including free energy, enthalpy, entropy, and specific heat, were obtained by performing phonon calculations at the optimized geometry.

The Helmholtz free energy *F*(*T*), entropy *S*(*T*), and heat capacity *C_v_*(*T*) with temperature can be expressed as follows [16]:

(2) $$F(T)=\frac{1}{2}\sum \hbar \omega +k_{Β}T\sum \ln[1-e^{-\beta \hbar \omega }]$$

(3) $$S(T)=-k_{Β}\sum \ln[1-e^{-\beta \hbar \omega }]-\frac{1}{T}\sum \frac{\hbar \omega }{e^{\beta \hbar \omega }-1}$$

(4) $$C_{V}(T)=k_{Β}\sum {\left(\beta \hbar \omega \right)}^{2}\frac{e^{\beta \hbar \omega }}{{\left[e^{\beta \hbar \omega }-1\right]}^{2}}$$

where $\beta ={\left(k_{Β}T\right)}^{-1}$, $k_{Β}$ represents the Boltzmann constant, T is the system temperature, and ℏω is the energy of individual phonon modes.

The Gibbs free energy is defined at a constant pressure as follows [35,36]:

(5) $$G=\underset{V}{\min}\left[U(V)+F(T,V)+pV\right]$$

where $\underset{V}{\min}\left[function\;of\;V\right]$ denotes the unique minimum of the bracketed expression with respect to the volume *V*, *U* is the total energy of the system, and *p* is the external pressure.

The isobaric heat capacity was subsequently derived through thermodynamic integration [37]:

(6) $$C_{p}=-T\frac{\partial^{2}G}{\partial T^{2}}$$

Raman intensities, corresponding to third-order derivatives of the total energy with respect to vibrational modes (atomic positions) and the applied laser field (electric field twice), were computed in CASTEP [26] by combining perturbation theory (second derivative with respect to the electric field) and finite differences (third derivative with respect to atomic displacements).

For polyhalite mineral, the temperature-dependent isobaric heat capacity derived from first-principles calculations was fitted to a Haas–Fisher-type polynomial using nonlinear least-squares regression [18]:

(7) $$C_{p}^{calc}=A_{1}+A_{2}T+A_{3}T^{-2}+A_{4}T^{-0.5}+A_{5}T^{2}$$

where *A*_1_, *A*_2_, *A*_3_, *A*_4_, and *A*_5_ are the coefficient of the polynomial *Cp*(*T*), and *T* is the system temperature.

$C_{p}^{calc}$ was determined through density functional theory (DFT) calculations in this study; the corresponding data fitting procedure and the resulting value of $C_{p}^{calc}$ are presented in Figure S4 of the Supplementary Materials.

It should be noted that the present thermodynamic calculations are based on the quasi-harmonic approximation (QHA). While QHA accounts for volume-dependent phonon frequencies, it neglects intrinsic anharmonic phonon–phonon interactions. At temperatures above 600 K, particularly approaching the phase transition range (620–800 K), anharmonic effects may become non-negligible. Consequently, the calculated heat capacity and entropy in this high-temperature regime may exhibit deviations from actual physical values, typically leading to a slight overestimation of Cp compared to experiments.

The enthalpy and Gibbs energy function were then computed using the equations [37]:

(8) $${\left(H_{T}-H_{298.15}\right)}^{calc}=\int_{298.15}^{T}C_{p}^{calc}(T)dT$$

(9) $${\left(G_{T}-G_{298.15}\right)}^{calc}={\left(H_{T}-H_{298.15}\right)}^{calc}-TS_{T}^{calc}$$

where $S_{T}^{calc}$ is the entropy. It was determined through density functional theory (DFT) calculations in this study; the corresponding data fitting procedure and the resulting value of $S_{T}^{calc}$ are presented in Figure S5 of the Supplementary Materials, calculated from first principles.

The enthalpies $\Delta_{f}H$ and free energies $\Delta_{f}G$ of the formation of polyhalite at different temperatures were obtained by means of the expressions [37]:

(10) $$\Delta_{f}H(T)=\Delta_{f}H^{0}+{\left(H_{T}-H_{298.15}\right)}^{calc}-\sum_{i}^{elements}n_{i}(H_{T}-H_{298.15}{)}_{i}^{\exp}$$

(11) $$\Delta_{f}G(T)=\Delta_{f}H(T)-T\left\{{S_{T}}^{calc}-\sum_{i}^{elements}n_{i}(S_{T}{)}_{i}^{\exp}\right\}$$

In the above equations, $\Delta_{f}H^{0}$ represents the standard formation enthalpy at 298.15 K and 1 bar, while ${\left(H_{T}-H_{298.15}\right)}_{i}^{\exp}$ and ${\left(S_{T}\right)}_{i}^{\exp}$ denote the enthalpy and entropy functions of constituent elements with their stoichiometric coefficients $n_{i}$. The enthalpy and entropy functions for the elements H, O, S, Ca, Mg, and K were taken from JANAF tables [37]. The formation reaction equilibrium constants were derived from the corresponding free energy values through the fundamental thermodynamic relation [37]:

(12) $$\Delta_{f}G(T)=-RTInK$$

where *K* is the equilibrium constant, $\Delta_{f}G\left(T\right)$ is the Gibbs free energy of formation at temperature *T*, and *R* is the universal gas constant (8.314 J·mol^−1^·K^−1^).

At the optimized geometry, the X-ray powder diffraction pattern of polyhalite was computed using the REFLEX, a module within the Material Studio Package, employing CuK_α_ radiation (λ = 1.540562 Å).

## 4. Conclusions

In this study, the thermodynamic and vibrational properties of polyhalite were systematically investigated using density functional theory (DFT). The calculated lattice parameters are in excellent agreement with experimental data, with a volume deviation of only 0.87%, compared to experimental data [20]. The computed Raman spectra reveal characteristic vibrational signatures arising from the free tetrahedral SO_4_^2−^ units within the polyhalite structure: a strong symmetric stretching mode (ν_1_) at 1024 cm^−1^, a symmetric bending mode (ν_2_) at 464 cm^−1^, and an anti-symmetric bending mode (ν_4_) at 627 cm^−1^. These calculated frequencies show remarkable consistency with experimental values [14,24], confirming the reliability of our computational approach. Thermodynamic properties, including the isobaric heat capacity, entropy, enthalpy, and Gibbs free energy, were obtained over the temperature range of 0–1000 K. At 298.15 K, the calculated isobaric specific heat and entropy are 420.72 and 531.39 J·mol^−1^·K^−1^, respectively, and the derived free energy of formation is −5670 kJ·mol^−1^.

## Figures and Tables

**Table 1:** Lattice parameters for polyhalite ($P\bar{1}$, Z = 1) from the DFT calculations at the GGA/PBE level of theory and available experimental data.

Parameters	a (Å)	b (Å)	c (Å)	α (°)	β (°)	γ (°)	Vol. (Å^3^)
This work	6.9142	6.5569	9.1895	102.48	104.81	107.05	365.488
Exp [20]	6.975	6.984	8.899	104.01	101.19	114.10	362.337
Cal.-DFT [16]	7.09	7.10	8.96	103.9	100.6	114.6	377.14

**Table 2:** Coefficients of the Hass–Fisher heat capacity polynomial C_p_(T) for polyhalite. Range of validity: 15–1000 K.

A_1_ × 10^−2^	A_2_ (T)	A_3_ × 10^−4^ (T^−2^)	A_4_ × 10^−3^ (T^−0.5^)	A_5_ × 10^4^ (T^2^)	R^2^
4.18392	0.66319	6.92945	−2.77001	−4.02279	0.99763

**Table 3:** Calculated and experimental Raman shifts, intensities, and assignations.

Calculated Raman Shift (cm^−1^)	Intensity (Å^4^)	Experimental Raman Shift [22,24] (cm^−1^)	Assignment
1196	40	1181	ν_3_ anti-symmetry stretching
1192	65	1165	
1169	134	1130	
1099	13	1094	
1066	18	1069	
1024	283	1014	ν_1_ symmetry stretching
972	215	987	
627	7	626	ν_4_ anti-symmetry bending
610	11	620	
464	13	464	ν_2_ symmetry bending
438	12	436	

**Table 4:** Calculated infrared shifts, intensities, and assignations.

Calculated Infrared Shift (cm^−1^)	Intensity (Å^4^)	Assignment
1196	22	ν_3_ anti-symmetry stretching
1161	48	
1090	40	
1062	45	
1027	18	ν_1_ symmetry stretching
972	2	
620	4	ν_4_ anti-symmetry bending
609	2	
468	3	ν_2_ symmetry bending
441	1	

## Data Availability

The original contributions presented in this study are included in the article/Supplementary Material. Further inquiries can be directed to the corresponding author(s).

## Supplementary Materials

The following supporting information can be downloaded at: https://www.mdpi.com/article/10.3390/molecules31081269/s1. Table S1. Polyhalite bond distances (in Å). Atom numbering follows the same convention as in a previous work. Table S2. Main reflections in the X-Ray powder spectrum of polyhalite. Table S3. Calculated isobaric heat capacity function of polyhalite. Temperature and heat capacity values are given in K and J mol^−1^·K^−1^ units, respectively. Table S4. Calculated entropy function of polyhalite. Temperature and entropy values are given in K and J·mol^−1^·K^−1^ units, respectively. Table S5. Calculated enthalpy function, H_T_-H_298.15_, of polyhalite. Temperature and enthalpy values are given in K and J·mol^−1^ units, respectively. Table S6. Calculated free-energy function, G_T_-G_298.15_, of polyhalite. Temperature and enthalpy values are given in K and J·mol^−1^ units, respectively. Table S7. Calculated isobaric heat capacity, entropy, enthalpy and free energy functions of polyhalite. The values of isobaric heat capacity and entropy are given in units of J·mol^−1^·K^−1^, and the values of enthalpy and free energy are given in units of kJ mol^−1^. Table S8. Calculated enthalpy (Δ*_f_*H) and free energy (Δ*_f_*G) of formation and Reaction constant (Log_10_K) of polyhalite as a function of temperature. The values of enthalpy and free energy are given in units of kJ mol^−1^. Figure S1. X-ray powder diffraction pattern of polyhalite obtained from the sample collected at Kunteyi Playa in Qinghai, China. Figure S2. CASTEP Thermodynamic Properties: Calculated heat capacity vs. temperature. Figure S3. CASTEP Thermodynamic Properties: Calculated entropy vs. temperature. Figure S4. Data fitting and coefficients of the heat capacity polynomial C_p_(T) for polyhalite. Range of validity: 15–1000 K. Figure S5. Data fitting and coefficients of the entropy polynomial S (T) for polyhalite. Range of validity: 15–1000 K.
