# Supplementary material for: New Insights into the Thermodynamic Properties and Raman Vibrational Modes of Polyhalite from Density Functional Theory
Source: Molecules. 2026 Apr 12;31(8):1269. doi: 10.3390/molecules31081269 (PMC13118967; doi:10.3390/molecules31081269)
Supplement: Supplementary file 1 [file molecules-31-01269-s001.zip › molecules-4215945-supplementary.pdf]

SUPPLEMENTARY MATERIALS

New Insights into the Thermodynamic Properties and Raman Vibrational Modes of Polyhalite from Density Functional Theory

Huaide Cheng 1,2,*, Yugang Chen 1,3 and Shichun Zhang 1,3

[1] Key Laboratory of Green and High-End Utilization of Salt Lake Resources, Qinghai Institute of Salt Lakes, Chinese Academy of Sciences, Xining 810008, China;
[2] Qinghai Provincial Key Laboratory of Geology and Environment of Salt Lakes, Xining 810008, China
[3] Qinghai Provincial Key Laboratory of Resources and Chemistry of Salt Lake, Xining 810008, China
* Correspondence: chenghuaide@isl.ac.cn

Table S1. Polyhalite bond distances (in Å). Atom numbering follows the same convention as in a previous work [1].

Bond	This work	Exp.[1]
S1-O3	1.467	1.461
S1-O5	1.614	1.482
S1-O2	1.479	1.484

S2-O6	1.458	1.461
S2-O8	1.483	1.472
S2-O7	1.498	1.473
S2-O1	1.500	1.504
Mg-O1	2.084	2.164
Mg-O2	2.342	2.045
Mg-O9	2.052	2.017
Ca-O1	2.643	2.479
Ca-O2	2.680	2.686
Ca-O3	2.293	2.413
Ca-O4	2.390	2.399
Ca-O5	2.541	2.554
Ca-O6	2.388	2.407
Ca-O7	2.482	2.609
Ca-O8	2.517	2.516
K-O1	3.281	3.164
K-O3	3.357	3.196
K-O6	2.882	2.998
K-O7	2.742	2.886
K-O8	2.822	2.787
K-O9	2.612	2.815

Table S2. Main reflections in the X-Ray powder spectrum of polyhalite.

**Exp.[2]**		**Exp.[1]**		**This work**	
2θ(deg)	I(%)	2θ(deg)	I(%)	2θ(deg)	I(%)
14.73	22.81	14.74	23.19	14.35	21.49
14.9	8.56	14.89	8.67	14.75	9.71
14.95	5.18	14.96	5.5	14.90	7.82

18.38	7.44	18.39	7.8	18.55	16.91
25.59	8.67	25.62	8.84	25.75	10.90
26.07	15.17	26.06	14.5	25.90	3.99
28.01	100	27.99	100	27.80	100
28.03	69.38	28.05	69.88	27.90	57.16
29.99	13.19	30.01	12.56	29.90	16.09
30.26	33.5	30.3	32.74	29.95	33.75
30.61	59.2	30.54	59.42	30.05	66.41
30.72	40.37	30.72	40.32	30.10	14.78
30.82	5.18	30.75	5.29	30.80	8.18
30.85	39.86	30.9	39.57	30.85	7.25
31.19	9.22	31.15	9.1	31.35	4.96
31.32	36.21	31.34	35.64	31.40	20.05
35.03	6.12	35.09	5.88	35.05	9.36
35.86	5.67	35.89	5.2	35.3	8.72
36.52	6.24	36.53	6.06	38.5	1.03
37.26	6.15	37.27	6.35	38.55	7.29
37.53	7.38	37.56	7.38	38.6	2.60
38.19	12.53	38.14	12.32	38.65	15.18
38.5	5.28	38.42	5.29	38.7	4.90
38.7	8.42	38.67	8.47	38.75	0.65
40.03	7.18	40.04	6.85	40.3	6.08
40.58	6.69	40.5	6.73	40.65	2.84
41.37	6.35	41.42	6.21	41.3	11.16
45.22	6.87	45.29	6.56	45.15	1.44
45.55	7.97	45.57	7.59	45.55	7.53
46.5	8.98	46.55	8.74	46.35	13.79
47.9	12.39	47.95	11.92	48.2	7.90
48.47	9.73	48.5	9.43	48.4	4.69
48.86	6.1	48.86	5.84	48.85	3.35
50.02	5.92	49.9	5.79	50.2	5.03
50.31	14.53	50.38	14.05	50.35	9.25
51.19	9.53	51.16	9.25	51.05	10.18
51.46	11.66	51.5	11.24	51.1	10.55
52.4	5.63	52.41	5.4	52.6	3.40
52.85	6.22	52.8	5.97	52.9	8.30
53.81	5.84	53.87	5.56	53.4	2.09

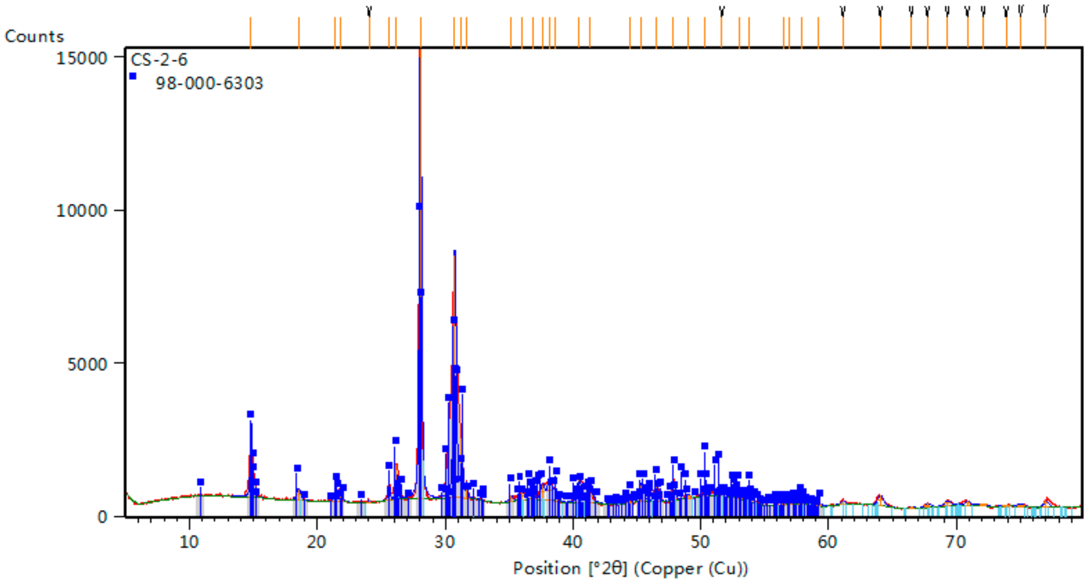

Pattern List

Ref.Code	Compound Name	Chem. Formula	Score	SemiQuant [%]
98-000-6303	Polyhalite	H4 Ca2 K2 Mg1 O18 S4	55	100

Figure S1. X-ray powder diffraction pattern of polyhalite obtained from the sample collected at Kunteyi Playa in Qinghai, China.

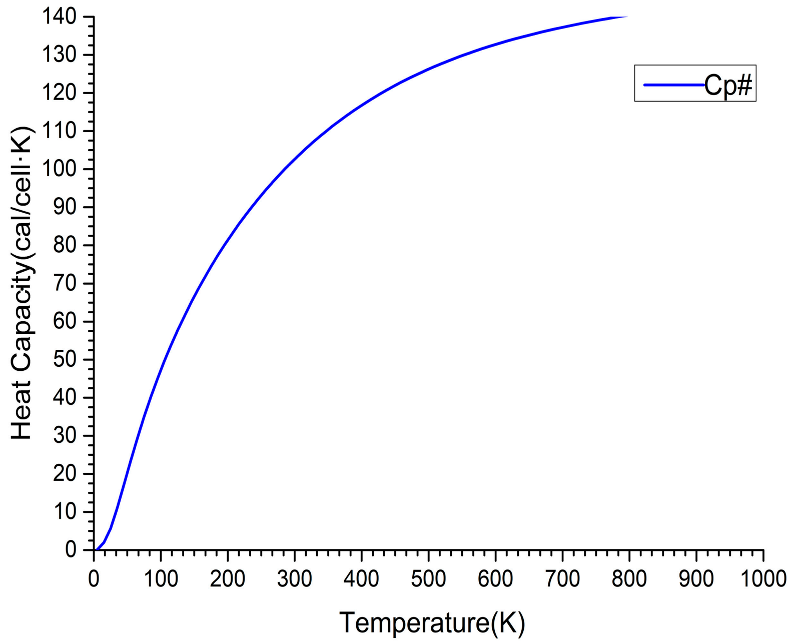

Figure S2. CASTEP Thermodynamic Properties: Calculated heat capacity vs. temperature

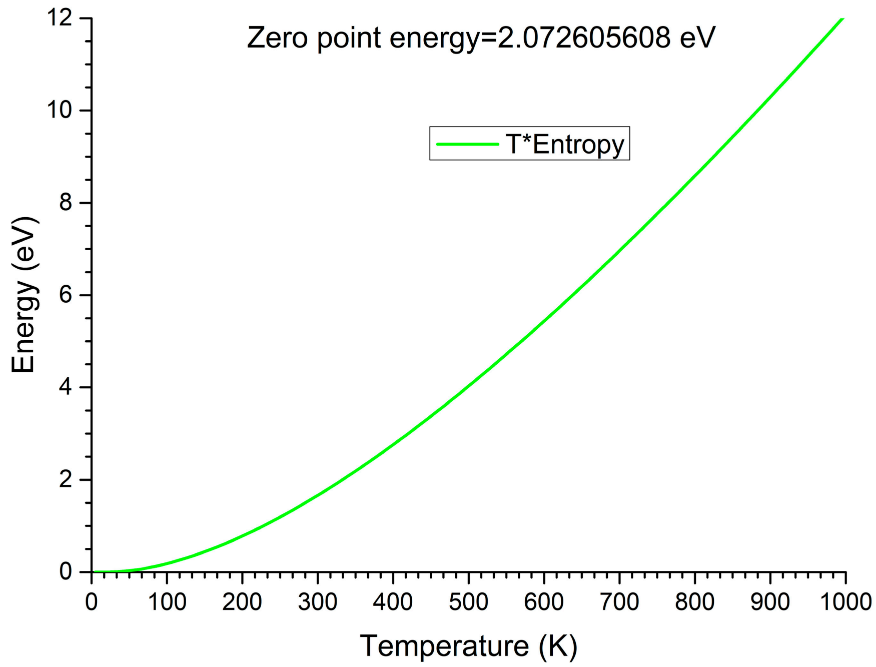

Figure S3. CASTEP Thermodynamic Properties: Calculated entropy vs. temperature

Table S3. Calculated isobaric heat capacity function of polyhalite. Temperature and heat capacity values are given in K and $J \cdot mol^{-1} \cdot K^{-1}$ units, respectively.

T	C_p	T	C_p	T	C_p
15.05051	8.16428	346.7172	460.00067	678.3838	570.66670
25.10101	23.77489	356.7677	465.86007	688.4343	572.34440
35.15152	46.49729	366.8182	471.48890	698.4848	573.96595
45.20202	72.40023	376.8687	476.89644	708.5354	575.53363
55.25253	98.57798	386.9192	482.09159	718.5859	577.04964
65.30303	123.60485	396.9697	487.08294	728.6364	578.51606
75.35354	146.99553	407.0202	491.87876	738.6869	579.93489
85.40404	168.71750	417.0707	496.48703	748.7374	581.30803
95.45455	188.91091	427.1212	500.91545	758.7879	582.63730
105.50505	207.75486	437.1717	505.17144	768.8384	583.92443
115.55556	225.41418	447.2222	509.26218	778.8889	585.17109
125.60606	242.02492	457.2727	513.19456	788.9394	586.37885
135.65657	257.69592	467.3232	516.97522	798.9899	587.54922
145.70707	272.51510	477.3737	520.61056	809.0404	588.68365
155.75758	286.55564	487.4242	524.10674	819.0909	589.78351
165.80808	299.88045	497.4747	527.46965	829.1414	590.85013
175.85859	312.54500	507.5253	530.70498	839.1919	591.88475
185.90909	324.59896	517.5758	533.81816	849.2424	592.88859
195.95960	336.08700	527.6263	536.81443	859.2929	593.86280
206.01010	347.04934	537.6768	539.69878	869.3434	594.80847
216.06061	357.52200	547.7273	542.47599	879.3939	595.72666
226.11111	367.53719	557.7778	545.15067	889.4444	596.61838
236.16162	377.12357	567.8283	547.72719	899.4949	597.48458
246.21212	386.30666	577.8788	550.20975	909.5455	598.32620
256.26263	395.10917	587.9293	552.60236	919.596	599.14412
266.31313	403.55140	597.9798	554.90885	929.6465	599.93918
276.36364	411.65156	608.0303	557.13287	939.697	600.71219
286.41414	419.42611	618.0808	559.27794	949.7475	601.46393
296.46465	426.89006	628.1313	561.34737	959.798	602.19514
306.51515	434.05717	638.1818	563.34437	969.8485	602.90653
316.56566	440.94021	648.2323	565.27196	979.899	603.59880
326.61616	447.55110	658.2828	567.13306	989.9495	604.27259
336.66667	453.90105	668.3333	568.93042	1000	604.92854

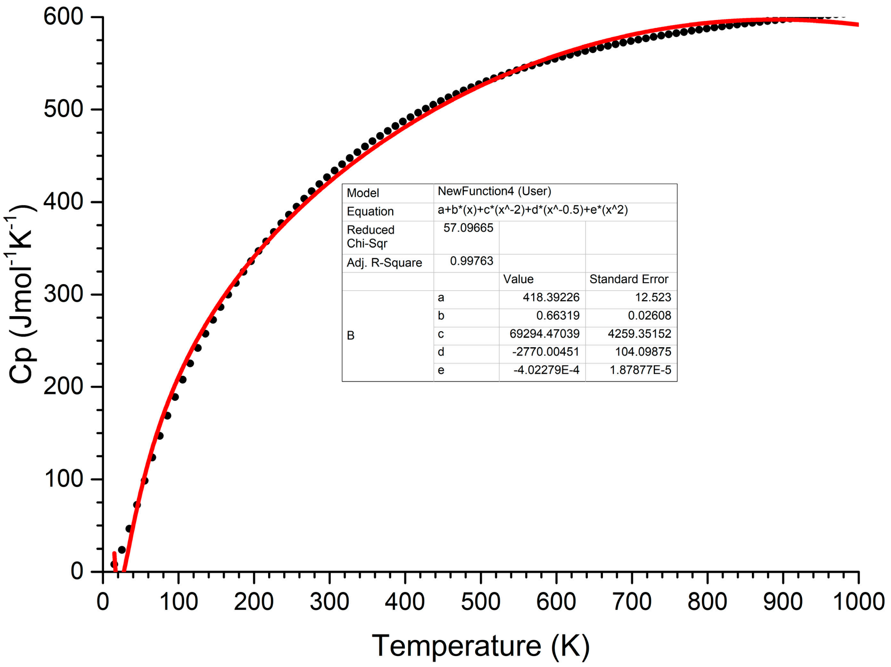

Model	NewFunction4 (User)		
Equation	a+b*(x)+c*(x^-2)+d*(x^-0.5)+e*(x^2)		
Reduced Chi-Sqr	57.09665		
Adj. R-Square	0.99763		
		Value	Standard Error
B	a	418.39226	12.523
	b	0.66319	0.02608
	c	69294.47039	4259.35152
	d	-2770.00451	104.09875
	e	-4.02279E-4	1.87877E-5

Figure S4. Data fitting and coefficients of the heat capacity polynomial $C_p(T)$ for polyhalite. Range of validity: 15–1000 K.

Table S4. Calculated entropy function of polyhalite. Temperature and entropy values are given in K and J·mol^{-1}·K^{-1} units, respectively.

T	S	T	S	T	S
15.05051	3.47717	346.7172	598.97501	678.3838	942.19664
25.10101	13.26191	356.7677	612.06962	688.4343	950.41035
35.15152	28.99916	366.8182	624.94628	698.4848	958.52721
45.20202	49.11725	376.8687	637.61142	708.5354	966.54925
55.25253	71.83423	386.9192	650.07117	718.5859	974.47846
65.30303	95.76334	396.9697	662.33132	728.6364	982.31675
75.35354	120.00433	407.0202	674.39742	738.6869	990.06600
85.40404	144.03659	417.0707	686.27475	748.7374	997.72804
95.45455	167.58840	427.1212	697.96838	758.7879	1005.30463
105.50505	190.53601	437.1717	709.48316	768.8384	1012.79750
115.55556	212.83787	447.2222	720.82375	778.8889	1020.20832
125.60606	234.49515	457.2727	731.99464	788.9394	1027.53873
135.65657	255.52958	467.3232	743.00014	798.9899	1034.79032
145.70707	275.97154	477.3737	753.84440	809.0404	1041.96462
155.75758	295.85389	487.4242	764.53145	819.0909	1049.06314
165.80808	315.20909	497.4747	775.06515	829.1414	1056.08735
175.85859	334.06788	507.5253	785.44926	839.1919	1063.03868
185.90909	352.45873	517.5758	795.68738	849.2424	1069.91850
195.95960	370.40786	527.6263	805.78303	859.2929	1076.72818
206.01010	387.93922	537.6768	815.73959	869.3434	1083.46902
216.06061	405.07469	547.7273	825.56034	879.3939	1090.14232
226.11111	421.83424	557.7778	835.24848	889.4444	1096.74933
236.16162	438.23613	567.8283	844.80708	899.4949	1103.29126
246.21212	454.29702	577.8788	854.23913	909.5455	1109.76930
256.26263	470.03219	587.9293	863.54753	919.596	1116.18460
266.31313	485.45561	597.9798	872.73509	929.6465	1122.53831
276.36364	500.58016	608.0303	881.80454	939.697	1128.83151
286.41414	515.41767	618.0808	890.75853	949.7475	1135.06529
296.46465	529.97907	628.1313	899.59963	959.798	1141.24068
306.51515	544.27448	638.1818	908.33034	969.8485	1147.35871
316.56566	558.31330	648.2323	916.95307	979.899	1153.42037
326.61616	572.10430	658.2828	925.47018	989.9495	1159.42665
336.66667	585.65564	668.3333	933.88397	1000	1165.37848

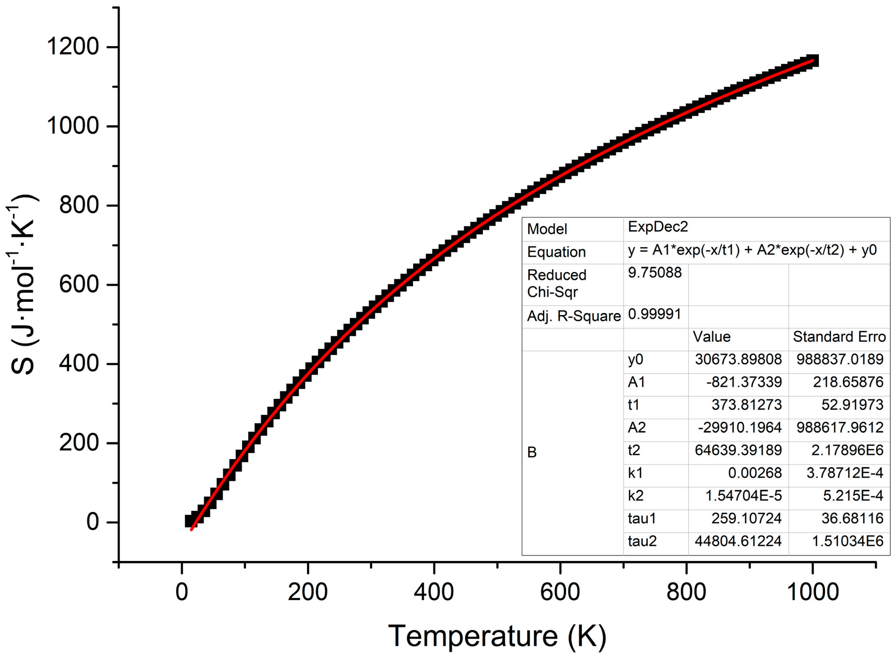

Figure S5. Data fitting and coefficients of the entropy polynomial S (T) for polyhalite.
Range of validity: 15–1000 K.

Table S5. Calculated enthalpy function, $H_T-H_{298.15}$, of polyhalite. Temperature and enthalpy values are given in K and J·mol^{-1} units, respectively.

T	H_T-$H_{298.15}$	T	H_T-$H_{298.15}$	T	H_T-$H_{298.15}$
15.05051	-74499.37223	346.7172	21205.58352	678.3838	195398.30001
25.10101	-74582.15918	356.7677	25776.05587	688.4343	201206.72269
35.15152	-74480.74140	366.8182	30404.98171	698.4848	207033.88932
45.20202	-73976.97700	376.8687	35090.72106	708.5354	212878.81832
55.25253	-73101.70090	386.9192	39831.68755	718.5859	218740.53391
65.30303	-71905.53231	396.9697	44626.34364	728.6364	224618.06580
75.35354	-70432.07220	407.0202	49473.19646	738.6869	230510.44894
85.40404	-68715.97606	417.0707	54370.79398	748.7374	236416.72329
95.45455	-66784.58729	427.1212	59317.72167	758.7879	242335.93357
105.50505	-64659.70530	437.1717	64312.59946	768.8384	248267.12903
115.55556	-62358.97165	447.2222	69354.07906	778.8889	254209.36327
125.60606	-59896.88678	457.2727	74440.84149	788.9394	260161.69403
135.65657	-57285.54716	467.3232	79571.59487	798.9899	266123.18301
145.70707	-54535.18293	477.3737	84745.07240	809.0404	272092.89571
155.75758	-51654.55473	487.4242	89960.03060	819.0909	278069.90123
165.80808	-48651.25066	497.4747	95215.24760	829.1414	284053.27217
175.85859	-45531.91171	507.5253	100509.52167	839.1919	290042.08444
185.90909	-42302.40514	517.5758	105841.66986	849.2424	296035.41713
195.95960	-38967.95966	527.6263	111210.52670	859.2929	302032.35239
206.01010	-35533.27192	537.6768	116614.94310	869.3434	308031.97532
216.06061	-32002.59149	547.7273	122053.78526	879.3939	314033.37381
226.11111	-28379.78928	557.7778	127525.93373	889.4444	320035.63846
236.16162	-24668.41311	567.8283	133030.28251	899.4949	326037.86248
246.21212	-20871.73336	577.8788	138565.73821	909.5455	332039.14155
256.26263	-16992.78067	587.9293	144131.21932	919.596	338038.57376
266.31313	-13034.37727	597.9798	149725.65550	929.6465	344035.25953
276.36364	-8999.16339	608.0303	155347.98694	939.697	350028.30148
286.41414	-4889.61943	618.0808	160997.16374	949.7475	356016.80436
296.46465	-708.08482	628.1313	166672.14535	959.798	361999.87501
306.51515	3543.22583	638.1818	172371.90008	969.8485	367976.62224
316.56566	7862.20897	648.2323	178095.40462	979.899	373946.15677
326.61616	12246.86012	658.2828	183841.64354	989.9495	379907.59117
336.66667	16695.26361	668.3333	189609.60894	1000	385860.03979

Table S6. Calculated free-energy function, $G_T-G_{298.15}$, of polyhalite. Temperature and enthalpy values are given in K and J·mol^{-1} units, respectively.

T	G_T-$G_{298.15}$	T	G_T-$G_{298.15}$	T	G_T-$G_{298.15}$
15.05051	-74551.70545	346.7172	-186469.33708	678.3838	-443772.67116
25.10101	-74915.04657	356.7677	-192590.59971	688.4343	-453088.40496
35.15152	-75500.10595	366.8182	-198836.67525	698.4848	-462482.84573
45.20202	-76197.17597	376.8687	-205205.05913	708.5354	-471955.49878
55.25253	-77070.72356	386.9192	-211693.32399	718.5859	-481505.90469
65.30303	-78159.16864	396.9697	-218299.12038	728.6364	-491133.63807
75.35354	-79474.82245	407.0202	-225020.17711	738.6869	-500838.30637
85.40404	-81017.28314	417.0707	-231854.30137	748.7374	-510619.54876
95.45455	-82781.66162	427.1212	-238799.37859	758.7879	-520477.03502
105.50505	-84762.21709	437.1717	-245853.37192	768.8384	-530410.46449
115.55556	-86953.56996	447.2222	-253014.32163	778.8889	-540419.56507
125.60606	-89350.89862	457.2727	-260280.34420	788.9394	-550504.09222
135.65657	-91949.81296	467.3232	-267649.63121	798.9899	-560663.82802
145.70707	-94746.18715	477.3737	-275120.44813	809.0404	-570898.58028
155.75758	-97736.03922	487.4242	-282691.13293	819.0909	-581208.18168
165.80808	-100915.46569	497.4747	-290360.09456	829.1414	-591592.48890
175.85859	-104280.61584	507.5253	-298125.81136	839.1919	-602051.38186
185.90909	-107827.68787	517.5758	-305986.82939	849.2424	-612584.76292
195.95960	-111552.93432	527.6263	-313941.76071	859.2929	-623192.55613
206.01010	-115452.66922	537.6768	-321989.28157	869.3434	-633874.70654
216.06061	-119523.27358	547.7273	-330128.13067	879.3939	-644631.17953
226.11111	-123761.19843	557.7778	-338357.10730	889.4444	-655461.96008
236.16162	-128162.96557	567.8283	-346675.06955	899.4949	-666367.05224
246.21212	-132725.16690	577.8788	-355080.93252	909.5455	-677346.47841
256.26263	-137444.46299	587.9293	-363573.66650	919.596	-688400.27884
266.31313	-142317.58172	597.9798	-372152.29520	929.6465	-699528.51105
276.36364	-147341.31710	608.0303	-380815.89404	939.697	-710731.24923
286.41414	-152512.52882	618.0808	-389563.58844	949.7475	-722008.58382
296.46465	-157828.14226	628.1313	-398394.55213	959.798	-733360.62090
306.51515	-163285.14916	638.1818	-407308.00553	969.8485	-744787.48179
316.56566	-168880.60869	648.2323	-416303.21416	979.899	-756289.30257
326.61616	-174611.64888	658.2828	-425379.48711	989.9495	-767866.23358
336.66667	-180475.46818	668.3333	-434536.17553	1000	-779518.43907

Table S7. Calculated isobaric heat capacity, entropy, enthalpy and free energy functions of polyhalite. The values of isobaric heat capacity and entropy are given in units of $J·mol^{-1}·K^{-1}$, and the values of enthalpy and free energy are given in units of $kJ·mol^{-1}$

T(K)	$C_p^{calc.}$	$S^{calc.}$	$(H_T-H_{298.15})^{calc.}$	$(G_T-G_{298.15})^{calc.}$
100	210.62	181.36	-65.85	-83.98
200	340.80	375.06	-37.60	-112.61
250	384.97	458.34	-19.42	-134.01
298.15	420.72	531.39	0.00	-158.43
300	421.99	534.07	0.78	-159.44
350	453.73	603.18	22.69	-188.42
400	481.24	666.50	46.08	-220.52
450	505.13	724.75	70.76	-255.38
500	525.82	778.57	96.54	-292.75
600	558.59	875.06	150.85	-374.18
700	580.95	959.60	207.91	-463.80
800	593.66	1034.97	266.72	-561.25

Table S8. Calculated enthalpy ($\Delta_f H$) and free energy ($\Delta_f G$) of formation and Reaction constant ($\text{Log}_{10}\ K$) of polyhalite as a function of temperature. The values of enthalpy and free energy are given in units of kJ·mol^{-1}

T(K)	$\Delta_f H$	$\Delta_f G$	$\text{Log}_{10}\ K$
100	-6098	-5960	3113
200	-6114	-5815	1519
250	-6118	-5740	1199
298.15	-6121	-5670	993
300	-6121	-5665	986
350	-6123	-5590	834
400	-6128	-5510	720
450	-6127	-5440	631
500	-6125	-5360	560
600	-6120	-5200	453
700	-6114	-5050	377
800	-6106	-4890	320

References

1. Bindi, L., Reinvestigation of polyhalite, $K_2Ca_2Mg(SO_4)_4.2H_2O$. *Acta Crystallographica Section E* **2005,** 61, (8), i135-i136.
2. Schlatti, M.; Sahl, K.; Zemann, A.; Zemann, J., Die Kristallstruktur des Polyhalits, $K_2Ca_2Mg[SO_4]_4.2H_2O$. *Tschermaks mineralogische und petrographische Mitteilungen* **1970,** 14, (2), 75-86.
